# Abundance and biofilm formation capability of *Vibrio cholerae* in aquatic environment with an emphasis on Hilsha fish (*Tenualosa ilisha*)

**DOI:** 10.3389/fmicb.2022.933413

**Published:** 2022-10-26

**Authors:** Subarna Sandhani Dey, Zenat Zebin Hossain, Humaira Akhter, Peter K. M. Jensen, Anowara Begum

**Affiliations:** ^1^Department of Microbiology, University of Dhaka, Dhaka, Bangladesh; ^2^BCSIR Laboratories Rajshahi, Bangladesh Council of Scientific and Industrial Research (BCSIR), Rajshahi, Bangladesh; ^3^Department of Public Health, School of Pharmacy and Public Health, Independent University, Dhaka, Bangladesh; ^4^Copenhagen Centre for Disaster Research, Institute of Public Health, University of Copenhagen, Copenhagen, Denmark

**Keywords:** cholera, aquatic environment, Hilsha fish, biofilm, persistence, cellular cytotoxicity, biofilm formation assay

## Abstract

The potentially deadly and sporadic diarrhea-causing agent, *Vibrio cholerae*, is present in a great number in the freshwater aquatic environment and can be transmitted to humans by different aquatic organisms. In the perspective of Bangladesh, an anadromous fish species Hilsha (*Tenualosa ilisha*) can act as a transmission vehicle of *V. cholerae* from the aquatic to the household kitchen environment. The present study was carried out to investigate the presence of *V. cholerae* in the aquatic habitat of Bangladesh with a major emphasis on freshly caught Hilsha fish, along with river water and plankton samples from the fish capture site. The study also detected the biofilm formation capability of *V. cholerae* within Hilsha fish that might help the transmission and persistence of the pathogen in aquatic habitat. Twenty out of 65 freshly caught fish (30.8%) and 1 out of 15 water samples (6.67%) showed the presence of *V. cholerae* and none of the plankton samples were positive for *V. cholerae*. The isolated strains were identified as non-O1 and non-O139 serogroups of *V. cholerae* and contain some major toxin and virulence genes. A few strains showed cellular cytotoxicity on the HeLa cell line. All strains were able to form biofilm on the microtiter plate and the detection of three genes related to biofilm formation (*vpsA*, *vpsL*, and *vpsR*) were also assayed using qPCR. In this study, the *in vitro* biofilm formation ability of the isolated strains may indicate the long-term persistence of *V. cholerae* in different parts of Hilsha fish. The abundance of *V. cholerae* only in freshly caught Hilsha fish and the absence of the pathogen in the surrounding aquatic environment could stipulate the role of Hilsha fish as one of the major transmission routes of *V. cholerae* from the freshwater aquatic environment of Bangladesh to the household kitchen environment.

## Introduction

*Vibrio cholerae* is a well-known human pathogen that causes severe watery diarrhea and dehydration and can lead to death if left untreated. Its role in causing and spreading infection has been investigated for over many years. *V. cholerae* is an indigenous member of the aquatic environment, including both marine, and freshwaters (Schuster et al., 2011). Its transmission from an aquatic environment to a healthy individual is studied widely and some studies proved that aquatic organisms, including planktons, can serve as a reservoir and vector of this pathogen (Colwell et al., 1985). Fishes that use plankton as a food source may also become a reservoir of *V. cholerae* (Halpern et al., 2008). In Bangladesh, intrusion of saline water from the Bay of Bengal into the river water of the coastal region during the dry season may influence the growth and transmission of *V. cholerae* (Lipp et al., 2002; Akanda et al., 2011; Hossain et al., 2018).

Cholera epidemics have been seen in many parts of the world, including Bangladesh, with seasonal regularity. Seasonal outbreaks of *V. cholerae* were found associated with planktonic and zooplanktonic blooms, which were influenced by water, temperature, the hour of sunlight, salinity, rainfall, sea surface height, etc. (Lobitz et al., 2000). In Bangladesh, two bimodal seasonal cholera peaks are observed in the early summer (March–May) and after the monsoon (September–November). Every year hundreds of cholera cases are originated from the southern coastal area of Bangladesh and this area has been considered an endemic region for cholera. It is assumed that aquatic environmental conditions (pH, salinity, plankton bloom, and water temperature) of the Bay of Bengal favor the growth and survival of epidemic strains of *V. cholerae* (Lipp et al., 2002; Huq et al., 2005; Akanda et al., 2011).

Fishes act as a natural reservoir and vector for *V. cholerae* (Halpern and Izhaki, 2017), and *V. cholerae* already has been isolated from marine fishes (Scheelbeek et al., 2009; Senderovich et al., 2010; Kumar and Lalitha, 2013), frozen fish stocks (Acharjee et al., 2012), guts of Zebrafish (Runft et al., 2014), intestines of Tilapia (Traoré et al., 2014), gills of Tilapia (Hounmanou et al., 2016), filets of processed *Pangasius* fish, and also from the water used to rinse them (Thi et al., 2014). In Japan, non-O1 strains of *V. cholerae* were identified from diseased Ayu fish, which is one of the important species of Japanese anadromous fish (Kiiyukia et al., 1992). A recent study by Hounmanou et al. (2019) showed that both toxigenic and non-toxigenic strains of *V. cholerae* are able to colonize in the intestine of Tilapia (Hounmanou et al., 2016) and Zebrafish (Runft et al., 2014) and horizontally transfer to naïve Tilapia fish. A previous study in Bangladesh revealed that Hilsha fish (*Tenualosa ilisha*), which are an inhabitant of the coastal region, can serve as a vector and reservoir for *V. cholerae* (Hossain et al., 2018).

The migration pattern of Hilsha (*Tenualosa ilisha)* is widely studied in the southeast region of Asia. Hilsha migrates from the Bay of Bengal for spawning (Hora, 1938; Pillay, 1958) during the monsoon, grows in freshwater, and returns to the ocean (Ahsan et al., 2014; Bhaumik, 2015; Miah, 2015). In Bangladesh, the spawning migration toward estuaries and rivers starts in July and ends in October (Bhaumik, 2015) and the peak period of breeding is from September to October (Miah, 2015). Due to their unique life cycle, Hilsha fish travel from the Bay of Bengal to freshwater rivers (Dutta and Hazra, 2017), and persistent bacterium within fish may also be transmitted to the freshwater environment.

*V. cholerae* persists in the aquatic environment of both endemic and non-endemic regions and this persistence is enhanced by the biofilm formation capability on various biotic and abiotic surfaces (Lutz et al., 2013). Biofilm helps the bacteria to stick with one another and also on a solid surface (López et al., 2010). For aquatic bacteria, surface attachment helps to access nutrients at the liquid-surface interface and thus helps the bacteria to survive in the nutrient-limited natural environment (Dawson et al., 1981). Examples of the biotic surfaces in the aquatic environment are ship hulls (Shikuma and Hadfield, 2010), zooplankton (Tamplin et al., 1990; Epstein, 1993; Huq et al., 2005; Turner et al., 2009), microalgae (Hood and Winter, 1997), and floating aggregates (Alam et al., 2006). In aquatic habitats, *V. cholerae* is capable of attaching to live copepods and their egg sacs (Huq et al., 1983), freshly harvested oyster samples from estuarine (Twedt et al., 1981), internal organs of fishes, for example, digestive tract (Alam et al., 2007; Senderovich et al., 2010), outer scale sample (Du Preez et al., 2010), etc. It has been also suggested that the association of *V. cholerae* with biotic surfaces increases the survival rate of *V. cholerae* in the aquatic environment (Huq et al., 1983).

Toxigenic strains of *V. cholerae* can cause explosive outbreaks when introduced into populations with poor sanitary infrastructure (O’Connor et al., 2011; Barzilay et al., 2013; Jackson et al., 2013). According to WHO, worldwide the annual mortality rate of cholera is 1.4–4.0 million cases with 21,000–143,000 deaths (World Health Organization [WHO], 2020). Bangladesh faces more than 100,000 cases of cholera each year (Ali et al., 2015). Major cholera outbreaks are caused by the O1 and O139 serogroups of *V. cholerae* containing major virulence factors like cholera toxin (CT) and toxin co-regulated pilus (TCP) (Nair et al., 1988; Pal et al., 1992; Chatterjee et al., 2009; Shin et al., 2011; Awasthi et al., 2013). Other serogroups collectively called non-O1/O139 are also able to cause cholera-like diarrhea, and other intestinal infections in humans (Dziejman et al., 2005; Deshayes et al., 2015). The virulence factors of non-O1/O139 *V. cholerae* are mostly cholera toxin independent but associated with different types of secretion systems, e.g., type six secretion system (T6SS) (Kostakioti et al., 2005), type III (T3SS) (Alam et al., 2011), heat-stable enterotoxin (NAG-ST), hemolysin (Honda and Finkelstein, 1979; Chatterjee et al., 2009), and cholix exotoxin (Awasthi et al., 2013).

The rationale of the current study was to investigate the transmission of *V. cholerae* from the Bay of Bengal to the freshwater habitat of Bangladesh *via* an aquatic reservoir. Previous studies from Bangladesh investigated the environmental transmission of *V. cholerae* in particular focusing on river water and plankton (Lipp et al., 2003; Ferdous et al., 2018; Rahman et al., 2018; Daboul et al., 2020). However, fish is an integral part of the aquatic environment, which is often not considered an important source of transmission vehicle of *V. cholerae*. In this study, the prevalence of *V. cholerae* is analyzed in freshly caught Hilsha fish along with surrounding river water and the plankton samples. As the environmental persistence capability of bacteria largely relies on biofilm formation, the biofilm formation ability of the isolated *V. cholerae* strains was examined and the virulence profile and cytotoxicity of isolated strains were also investigated. The study was conducted as an extension part of a research project funded by the Danish Government (DANIDA) called “Combating Cholera Caused by Climate Change” (C5).^1^

## Materials and methods

### Sample collection and processing

**FIGURE 1:**
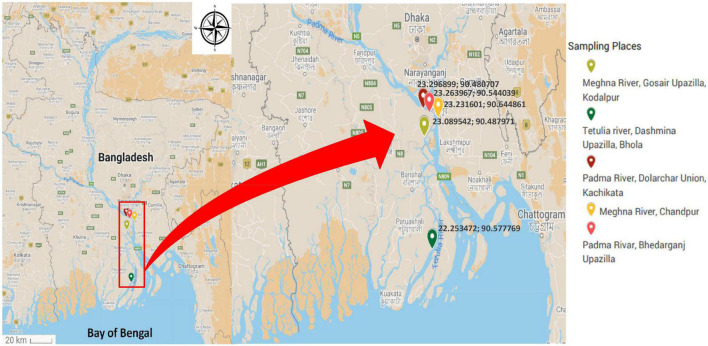
Map showing the sampling site of freshly caught Hilsha fish.

The sampling places have been chosen depending on the spawning location of Hilsha fish, which were the estuary points near the bank of Padma, originating from the Ganges river and Meghna river (Ahsan et al., 2014). From June 2016 to April 2017, 65 fish, 15 water, and 9 plankton samples were collected from five different river locations in Bangladesh. Information on sampling locations is listed in Table 1 and Figure 1. Each fish was collected from fishermen in an individual sterile collection bag and transported directly to the laboratory in the Department of Microbiology, the University of Dhaka within 4 h of collection in a cool box maintaining cold condition.

**TABLE 1:** Sampling locations and number of collected fish, water, and plankton samples in the presence (%) of the *ompW* gene.

Sampling places	G.P.S coordinates	No. of fish caught (no. of *ompW* positive fish)	No. of water sample collected (no. of *ompW* positive water)	No. of plankton sample collected (no. of *ompW* positive Plankton)
Meghna river, Gosair Upazilla, Kodalpur	23.089542^°^N 90.487971^°^ E	10 (9)	0	0
Tetulia river, Dashmina Upazilla, Bhola	22.253472^°^N 90.577769^°^E	08 (0)	3 (0)	3 (0)
Padma river, Dolarchar Union, Kachikata	23.296899^°^N 90.480707^°^E	10 (3)	3 (0)	3 (0)
Meghna river, Chandpur	23.231601^°^N 90.644861^°^E	10 (0)	1 (0)	1 (0)
Padma river, Bhedarganj Upazilla	23.263967^°^N 90.544039^°^E	15 (4)	8 (1)	2 (0)
Meghna river, Gosair Upazilla, Kodalpur	23.089542^°^N 90.487971^°^E	12 (4)	0	0

Four different body parts of fish were taken as samples, they are the outer surface, gill, gut, and rectum, and enriched in alkaline peptone water (APW) overnight. For the river water sample, 100 ml of water was collected in sterile polypropylene bottles from two or three different locations at each sampling site. The water samples were filtered through a 0.22 μm membrane filter and the filter paper was placed in APW for enrichment. In the case of the plankton sample, 100 ml of water was collected from the edge of the river and passed through a 0.22 μm pore size plankton net (Huq et al., 2012). The sample was filtered through a 0.22 μm membrane filter and the filter paper was placed within a stomacher bag with normal saline, and then homogenized in a stomacher (Seward Stomacher 80, Lab UK) and 1 mL of the homogenized sample was enriched in APW.

All overnight enriched samples were further assessed by polymerase chain reaction (PCR) for the presence of the *ompW* gene specific to *V. cholerae* (Nandi et al., 2000; Choopun et al., 2002; Huq et al., 2012). A total of 284 samples were taken from fish, water, and plankton. Their Total deoxyribonucleic acid (DNA) was extracted using a previously published protocol (De Medici et al., 2003). Only the *ompW-*positive samples were cultured in thiosulfate citrate bile salt sucrose (TCBS) agar (Oxoid, UK). The yellow, shiny colonies were picked from the culture plate and further confirmed by the biochemical and molecular tests.

### Detection of *Vibrio cholerae* in total deoxyribonucleic acid by polymerase chain reaction

The boiled template method (De Medici et al., 2003) was followed for the extraction of total DNA. In total DNA, the presence of *V. cholerae* was confirmed by PCR using two primers for the outer membrane protein (*ompW*) gene (Nandi et al., 2000) (F-CACCAAGAAGGTGACTTTATTGTG and (R- GTTTGTCGAATTAGCTTCACC). PCR reactions were conducted according to the protocol used in by Hossain et al. (2018), and PCR product sizes (304 bp) were estimated using 100 bp DNA size markers (Invitrogen, USA).

### Serotyping of isolated *Vibrio cholerae* strains

For serotyping of isolated strains, the agglutination test was performed on clean glass slides using polyvalent O1 and 139 antisera (Denka Seiken, Japan).

### Molecular characterization of total deoxyribonucleic acid and *Vibrio cholerae* strains

#### Real-time detection of *ctxA* gene in outer membrane protein-positive total deoxyribonucleic acid extract

The sequences of oligonucleotide primers were sense (5′-TTTGTTAGGCACGATGATGGAT-3′) and anti-sense (5′-AC CAGACAATATAGTTTGACCCACTAAG-3′), which generate an 84 base pair amplicon within the *ctxA* gene. 5′ nuclease probe within the *ctxA* gene (TGTTTCCACCTCAATTAGT TTGAGAAGTGCCC) 5′ labeled with either FAM or TET and a Black Hole Quencher 1 on the 3′ end (Integrated DNA Technologies) (Tag Copenhagen A/S, Denmark) was used. The detection of the *ctxA* gene was assayed using a protocol previously described by Blackstone et al. (2007). The qPCR thermal cycling was run on ABI StepOne System (Life Technologies, USA) using an initial UNG incubation step at 50°C for 2 min and a polymerase activation step at 95°C for 10 min followed by 40 cycles of denaturation at 95°C for 15 s and a combined anneal and extension step at 60°C for 1 min. The other parameters of the machine were set as default for analysis.

#### Presence of other virulence-related genes in total deoxyribonucleic acid and *Vibrio cholerae* strains

Both *V. cholerae* positive total DNA and isolated strains were tested for major toxin and regulatory genes of *V. cholerae* by performing PCR. The primers used in this study are listed in Supplementary Table 2. The DNA extraction and PCR protocol are described in the previous section.

### Cytotoxicity test

*V. cholerae* strains were grown under shaking conditions for 18 h in Trypticase soy broth (TSB; Difco, Detroit, Mich.) supplemented with 0.6% yeast extract (TSB-YE). The culture supernatant was collected by centrifugation and by passing it through a 0.22 μm pore size filter unit (corning incorporated, Germany), which made it a cell-free culture. The cell-free culture filtrate was taken in sterile test tubes that were kept at 4°C until they were used. HeLa cells were grown as monolayers in the Dulbecco’s minimum essential medium (Nissui Pharmaceutical Co., Ltd., Tokyo, Japan) supplemented with 10% (vol/vol) horse serum (Gibco Laboratories, Grand Island, N.Y.). Cell lines were maintained in 25-cm^2^ cell culture flasks (NUNC, Roskilde, Denmark) at 37°C supplemented with 5% CO_2_. A confluent monolayer of HeLa cells grown for 3–4 days was removed from the cell culture flasks, and 200 μl of the cell suspension (ca. 6.4 × 10^3^ cells) was added to each of the 96-well plates along with 50 μl of the cell-free culture filtrate, and the plates were incubated as described above. Morphological changes in HeLa cells were recorded at 24 h. The un-inoculated culture medium was used as medium control (Sharma et al., 1998).

### Determination of salt tolerance

The isolated strains were tested for their ability to grow at different NaCl concentrations of 0, 3, 5, 6, 8, and 10% in nutrient broth (lab lemco 1 g/L, yeast extract 2 g/L, and peptone 5 g/L) and turbidity (indicates growth) was scored spectrophotometrically at 600 nm wavelength after 24 h.

### Determination of antibiotic susceptibility

Antibiotic susceptibility of the *V. cholerae* strains was conducted by the agar disk diffusion method (Biemer, 1973) using commercial disks (Oxoid, UK). The strains were tested for Tetracycline 30 μg, Sulfamethoxazole-trimethoprim 25 μg, Chloramphenicol 30 μg, Ciprofloxacin 5 μg, Kanamycin 30 μg, Neomycin 30 μg, Oxytetracyclin 30 μg, Gentamicin 10 μg, Ampicillin 10 μg, Ceftriaxone 30 μg, Aztreonam 30 μg, and Nalidixic acid 30 μg, and were used in this study according to the standard guidelines of the Clinical and Laboratory Standards Institute (CLSI, 2013 January) (Patel et al., 2014). The zone standards for Enterobacteriaceae were used when there were no established breakpoint interpretive criteria for *V. cholerae*. *E. coli* ATCC 25922 was used as a quality control strain. The experiment was done in duplicate.

### Determination of biofilm formation

#### Biofilm formation ability on a microtiter plate

For the biofilm formation assay, the isolated bacterial strains along with the strain N-16961 (as a positive control) were assayed both qualitatively and quantitatively for biofilm formation in the microtiter plate (O’Toole, 2011). One replicate for each strain was taken and cell absorbance was measured in 595 nm wavelength. Here, sterile media was taken as the negative control, and OD cut-off value was calculated using the average OD of the negative control + 3 × standard deviation of the negative control. The OD cut-off value was separately calculated for each of the strains and biofilm formation by isolates was calculated and categorized according to the absorbance (Samadi et al., 2019).

#### Detection of biofilm formation-related genes in *Vibrio cholerae* strains by real-time polymerase chain reaction

Detection of the three genes of interest, *vpsR, vpsA*, and *vpsL*, was conducted by real-time PCR (Yildiz and Schoolnik, 1999). The sequence of the primers used for real-time PCR is listed in Table 2. The formula of the reaction mixture and cycling conditions were optimized for the detection of *vps* genes as per supplier instruction. In short, a 25 μl reaction mixture contains 12.5 μl 2X SYBR Green Universal Master Mix (Applied Biosystems, USA, containing AmpliTaq Gold^®^ DNA Polymerase, dNTPs, ROX Passive Reference, Uracil-N glycosylase), 2.5 μl of 100 nM of each primer, and 2.5 μl of DEPC-treated water (Carl Roth, Germany) with 5 μl of the template. The qPCR thermal cycling was run on the ABI StepOne System (Life Technologies, USA) with the following condition: 5 min at 95°C for the activation of Taq polymerase followed by 40 cycles of 30 s at 95°C and a combined annealing and extension step at 60°C for 1 min. DNA amplifications were observed from the fluorescent signal emitted for binding of the SYBR Green dye with double-stranded DNA.

**TABLE 2:** Sequences of the primers and amplicon size (bp) in the qPCRs used for the detection of biofilm formation gene *vps*.

Primer name	Sequence	Amplicon size (bp)	References
*VpsA*	F-TTCCCCTTGGCCTGAAGAG	67	Yildiz et al., 2001
	R-AGGTGCAAAGTGGTACTGCGT		
*vpsL*	F-ATCGCACCATAGTGAATCGCT	69	Yildiz et al., 2001
	R-CTGTGCCCATCCAGTAATGC		
*VpsR*	F-GTCTCAGCTCGATCTTCCCAA	63	Yildiz et al., 2001
	R-CGTTCCCGAATGCTTTTCAG		

### Statistical analysis

Both exploratory and regression models were used to analyze the data. To explore the influencing factor of different sample types on *V. cholerae* presence, a logistic regression analysis was performed in the presence of the *ompW* gene as the dependent variable and the type of samples from different sampling locations as the independent variable. The odds ratio or Exp(B) was considered under a 95% confidence interval.

In an exploratory analysis, different salt tolerance levels were compared with virulence factors. The absorbance of positive control strain N-16961 at different salt concentrations was considered as a cut-off value to categorize isolated 29 *V. cholerae* strains (absorbance more than cut-off value = 1 and absorbance less than cut off value = 0). Statistical analysis was carried out using SPSS (IBM SPSS Statistic 22) and results were measured as significant for *p*-value < 0.05.

## Results

### Prevalence of *Vibrio cholerae* in fish, river water, and plankton samples

**FIGURE 2:**
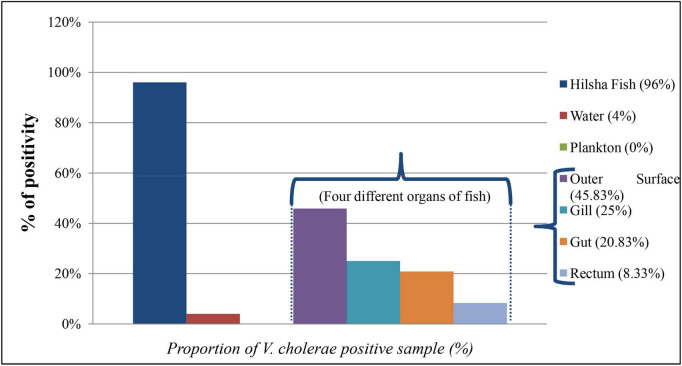
Proportion (%) of *V. cholerae*-positive direct DNA samples from fish, water, plankton, and different organs of fish (outer surface, gill, rectum, and gut).

A total of 284 DNA were extracted from 65 fish, 15 water, and 9 plankton samples. Among them, 25 samples were positive (8.80%) for the presence of the *ompW* gene in PCR, which is specific for *V. cholerae* outer membrane protein. Of all samples, 20 fishes (30.8%) and only one (6.7%) water samples were positive for *V. cholerae* in total DNA, but no *V. cholerae* was found in the plankton sample. The highest number of *V. cholerae*-positive Hilsha fish (9 out of 10 fish) was collected from Meghna river, Gosair Upazilla, and Kodalpur than other sampling points. From Tetulia river, Dashmina Upazilla, Bhola, and Meghna river, and Chandpur sampling points, all the samples were found negative for *V. cholerae.* From four different organs of all collected fish, 11 out of 65 (16.92%) total DNA from outer surface samples were positive for the presence of the *V. cholerae-*specific *ompW* gene, which was higher than the other three organs, and only 2 out of 65 (3.08%) total DNA were positive in case of the gut samples (Figure 2). Twenty-nine *V. cholerae* strains were isolated from the *ompW*-positive different organ parts of fish samples. No strains were isolated from river water and plankton samples (Figure 2).

### Serotyping of isolated *Vibrio cholerae* strains

All the strains (*n* = 29) with typical biochemical behaviors were subjected to serotyping for “O” antigen using the O1 polyvalent and O139 antisera. The strains did not show a typical serological reaction like that of control strains.

### Molecular characterization of total deoxyribonucleic acid and *Vibrio cholerae* strains

#### Real time detection of *ctxA* gene in outer membrane protein -positive total deoxyribonucleic acid extract

All the total DNA samples (*n* = 24) have shown negative results for the presence of the *ctxA* gene.

#### Presence of other virulence related genes in total deoxyribonucleic acid and *Vibrio cholerae* strains

**FIGURE 3:**
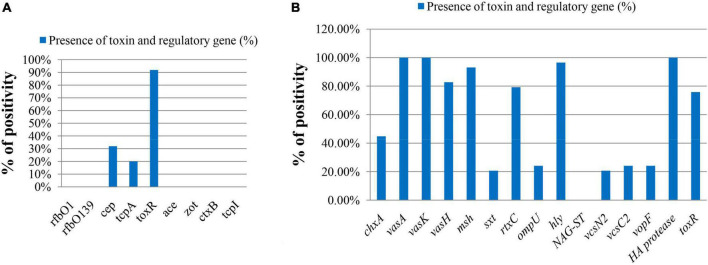
The molecular pattern of *ompW*-positive sample on the basis of toxin and regulatory genes of *V. cholerae.* **(A)** Direct DNA sample (% bar chart); **(B)** DNA sample of confirmed isolated *V. cholerae* strains (% bar chart).

The major virulence-related genes of *V. cholerae* like *rfbO1, rfbO139, ctxA, ctxB, ace, zot*, and *tcpI* were absent in both total DNA and isolated strains (Figure 3A). The spotting of the *tcpA* and *cep* genes was confirmed in 20.0% (5 out of 25) and 32.0% (8 out of 25), respectively, in *ompW*-positive total DNA samples. Other virulence-related genes were also present in *V. cholerae* strains, which are represented in Figure 3B. All strains contained *vasA, vasK*, and *HA protease* genes. The genes for type 3 secretion system (T3SS), *vcsN2*, *vcsC2*, and *vopF*, were also detected in 20.69%, 24.14%, 24.14% of strains, respectively. There is a pattern in the virulence genes among samples from different sampling points. *tcpA* and *cep* genes were present in total DNA extracted from two sampling sites Padma River, Dolarchar Union, Kachikata, and Padma River, Bhedarganj Upazilla. Other major virulence genes like *vcsN2, and vcsC2, vopF* were present mostly in the *V. cholerae* strains isolated from Meghna River, Gosair Upazilla, and Kodalpur. Both *sxt* and *chxA* were present mostly in the *V. cholerae* strains isolated from Meghna River, Gosair Upazilla, Kodalpur, and Padma River, Dolarchar Union, Kachikata.

### Cytotoxicity test

In the cellular cytotoxicity test, four strains, EL-1(b), EL-98(b), EL-187(b), and EL-238(b), were selected depending on the maximum presence of toxin and virulence genes. EL-1(b) and EL-187(b) strains showed 95% killing of cells, which is higher than the clinical strain of *V. cholerae* N-16961 (70–80%).

### Different salt concentration stress

The average highest cell growth was observed in 3% salt concentration (OD 0.328) and the lowest in 10% salt concentration (O.D. 0.0169). But strain EL-8(b) gave the highest optical density at 5% salt concentration (OD 0.737). The clinical isolate positive control N-16961 showed the highest optical density at 6% salt concentration (OD 0.468) and lowest at 0% salt concentration (OD 0.009) (listed in Supplementary Table 3).

### Antibiotic susceptibility assay

Antibiotics were selected according to their mode of action and frequency of use. The antibiotic response of the strains revealed that all were uniformly susceptible to Ceftriaxone and Aztreonam (100%). There were 9, 4, 3, and 2 strains out of a total of 29 strains that showed resistance to Ampicillin, Sulfamethoxazole-trimethoprim, Nalidixic acid, Neomycin, and Streptomycin, respectively. Five isolates showed intermediate resistance to Streptomycin, three to Ciprofloxacin, Neomycin, and Oxyteracyclin, and four to Neomycin.

### Biofilm formation assay

The results of the biofilm formation assay in the microtiter plate showed that all strains created a thin purple ring in the microtiter plate wells, which was an indication of the biofilm formation zone. All strains showed higher mean absorbance than the clinical reference strain *V. cholerae* O1 N16961. The lowest mean absorbance for the *V. cholerae* strains was 0.19 and the highest mean absorbance for the *V. cholerae* strains was O1 N16961, whereas the mean absorbance for *V. cholerae* N1T was 0.16. The mean absorbance of biofilm formation is listed in Supplementary Table 4. Classification of 29 strains for biofilm formation ability on microtiter plate was done according to Samadi et al. (2019). In this study, 41.38% (12 out of 29) of strains were strong, 44.83% (13 out of 29) of strains were moderate, and 13.79% (4 out of 29) of strains were weak biofilm former, and the clinical reference strain V. cholerae O1 N16961 was found as a weak biofilm former in our study.

Measuring the presence of three biofilm-formation-related genes, using real-time PCR, revealed that 25 strains of *V. cholerae* had CT values lower than 20 in the case of *vpsR*, which is the main regulatory gene for VPS synthesis. The other two structural genes for biofilm formation *vpsA* and *vpsL* were also present among the strains. The number of strains showed CT values lower than 20 for *vpsA* and *vpsL* genes, respectively, 19 and 22 out of 29 strains. Only two strains EL-195(a) and EL-195(b) showed the CT value higher than 35 for *vpsA* and *vpsL* gene, which indicates the absence of vpsA and vpsL gene in this two strains. But they contained the *vpsR* gene (CT value: 21.3 and 17.8, respectively) (listed in Supplementary Figure 1) and also formed a purple zone on the microtiter plate.

### Statistical analysis

The logistic regression analysis showed that the odds of *V. cholerae* presence in water is 0.16 times lower than Hilsha fish. Regarding plankton samples, the odds were undetermined (0.00).

In the Chi-square test, the test of independence implied there is no significant association exists between most of the virulence genes and salt tolerance (as the *p* > 0.05). However, few exceptions were also observed here, for example, the *msh* gene showed significant relation with both 5% (*p* = 0.011) and 10% (*p* = 0.008) salt tolerance, whereas *rtxC* showed a significant relationship with 0% salt tolerance (*p* = 0.033) and *ompU* showed with 3% salt tolerance (*p* = 0.002) (listed in Table 3).

**TABLE 3:** Chi-square test result (*p*-value) of virulence gene and different salt concentrations.

Virulence gene															
Salt concentration	*chxA*	*vasA*	*vasK*	*vasH*	*msh*	*sxt*	*rtxC*	*ompU*	*hlyB*	*NAG-ST*	*vcsN2*	*vcsC2*	*vopF*	*HA protease*	*toxR*
0%	0.088	−	−	0.414	0.626	0.543	0.033*	0.783	0.735	−	0.314	0.783	0.783	−	0.666
3%	0.626	−	−	0.414	0.626	0.156	0.361	0.002*	0.735	−	0.666	0.783	0.783	−	0.314
5%	0.922	−	−	0.513	0.011*	0.626	0.464	0.440	0.786	−	0.419	0.377	0.377	−	0.419
6%	0.796	−	−	0.729	0.301	0.197	0.333	0.144	0.472	−	0.542	0.243	0.243	−	0.760
8%	0.051	−	−	0.414	0.626	0.543	0.361	0.783	0.735	−	0.314	0.271	0.271	−	0.666
10%	0.526	−	−	0.177	0.008*	0.666	0.517	0.068	0.575	−	0.708	0.398	0.398	−	0.518

*p-values were significant (<0.05).

## Discussion

In our study, we investigated the prevalence of *V. cholerae* on aquatic surfaces to define the important transmission source of *V. cholerae* in a freshwater environment. In Bangladesh, the freshwater habitat is a possible main source of *V*. *cholerae* (Faruque et al., 1998; Ferdous et al., 2018) and studies showed that fishes are one of the intermediate reservoirs of *V. cholerae* in various aquatic ecosystems (Halpern and Izhaki, 2017; Hossain et al., 2018). A recent study on Tilapia fish was done in a laboratory to prove that fish act as a reservoir host of *V. cholerae* O1 El Tor strain and showed the survival and transmission of the pathogen horizontally to other fish (Hounmanou et al., 2019). And another recent study showed the population dynamics and virulence profile of *V. cholerae* strains isolated from freshly caught and local market Hilsha fish of Bangladesh and tried to find out the missing link between environmental and household contamination in Bangladesh (Hossain et al., 2018). The current study is an extension work of Hossain et al. (2018), and here, we have focused on the aquatic environmental transmission of *V. cholerae* with particular emphasis on Hilsha fish. We have detected *V. cholerae* in freshly collected fish along with surrounding river water and plankton samples. Our aim was to validate Hilsha fish as *V. cholerae* transmission vehicle in the riverine environment of Bangladesh. Hence, the abundance of *V. cholerae* within different parts of Hilsha has been investigated and the biofilm formation ability of the isolated strains was also studied.

In our study, we found *V. cholerae* attached to the outer surface, gill, gut, and rectum of freshly caught Hilsha fish (Figure 2). Also, 6.7% of the collected river water samples showed the presence of *V. cholerae* in PCR. People who live near the river area can be infected with *V. cholerae* while using river water for cleaning cloths, other utensils, and bathing. Previous reports revealed the incidence of cholera cases between 2016 and 2017 near Narayonganj, Matlab, and Mathbaria areas coinciding with the duration of our sampling period and close to our sampling points (Khan et al., 2020; Sack et al., 2021). The molecular profiling of both extracted total DNA and isolated strain DNA revealed that major virulence related genes like *tcpA* and *cep* are present in extracted total DNA, collected from different parts of Hilsha fish but they are not present in isolated *V. cholerae* strains. This is due to the tendency of *V. cholerae* to remain in VBNC (Viable But Non-Culturable State) or dormant state in the environment (Huq et al., 1990; Alam et al., 2007; Du Preez et al., 2010; Bhuyan et al., 2016).

In our study, all 29 strains isolated from fish samples were non-O1/non-O139 and negative for *ctx* and *zot* genes, which are the major virulence factors for O1 and O139 *V. cholerae.* However, the isolated strains contain other virulent genes like *vasA* (100%), *vasK* (100%), *vasH* (82.76%), *vcsN2* (20.69%), *vcsC2*, and *vopF* (24.14%), and *chxA* (44.83%) that are associated with cholera or cholera-like diarrhea by *ctx*-independent mechanism (Park et al., 2004; Dziejman et al., 2005; Kostakioti et al., 2005; Alam et al., 2011; Shin et al., 2011; Awasthi et al., 2013). In *V. cholerae*, genes comprising the virulence-associated (VAS) clusters, namely, *vasH, vasK, vasF*, and *vasA*, have been suggested to be of structural importance and critical for the Type Six Secretion System (T6SS) machinery (Kostakioti et al., 2005). Type Three Secretion System (T3SS) contains *vcsN2*, *vcsC2*, and *vopF*, and *vcsN2* is an ATPase and *vcsC2* is an inner-membrane protein. The domain structure and conservation of *vopF* suggested that it is a putative effector for the *V. cholerae* T3SS and likely to interact with the host actin cytoskeleton (Shin et al., 2011). Cholix toxin (ChxA) is a recently discovered exotoxin in *V. cholerae* that has been characterized as a third member of the eukaryotic elongation factor 2-specific ADP-ribosyltransferase toxins, and in a recent study, it is observed that *chxA is* an important virulence factor among non-O1/non-O139 *V. cholerae* strains (Awasthi et al., 2013).

Four selected strains showed a cytotoxic effect on HeLa cells though they lacked *ctxA* or *tcp* genes. They contained other toxin genes along with that hemolysin toxin (encoded by the *hlyA* gene) and previous evidence showed that hemolysin toxin contains cytolytic activity when demonstrated on Vero and other mammalian cells in culture (Honda and Finkelstein, 1979; Chatterjee et al., 2009). Average highest cell growth was observed in 3% salt concentration (OD 0.328) (Supplementary Table 3), which is much closer to seawater salinity (3.5%) (United States Geological Survey [USGS], 2020) and much higher than freshwater salinity (less than 0.1%) (United States Geological Survey [USGS], 2020).

*V*. *cholerae* biofilm could play a critical role in pathogenesis and disease transmission as they are more resistant to a stressful condition in the host and requires a lower infectious dose (Kostakioti et al., 2013; Silva and Benitez, 2016). It has been reported that surface colonization and biofilm formation enhance the persistence of *V*. *cholerae* in the environment and biofilm-encased cells are protected from predation and starving, as VPS synthesis allows the exploitation of attached surface nutrients (Matz et al., 2005; Lutz et al., 2013). Data also showed that persister cells of *V. cholerae* could survive in nutrient-poor “filter sterilized” lake water (FSLW) for about 700 days, these persister cells had a cell-to-cell aggregation and biofilm formation ability (Jubair et al., 2014). From the analysis of mean absorbance data of biofilm assay, it has been found that all of the strains showed positive *in vitro* biofilm formation in a microtiter plate. The presence of three major genes in all isolates behind the regulation (*vpsR*) and formation (*vpsA* and *vpsL*) of biofilm was quite remarkable in our study.

## Conclusion

In conclusion, tracking the possible route of *V. cholerae* dissemination to fresh water is crucial in a densely populated country like Bangladesh. The absence of *V. cholerae* in surrounding river water and plankton samples reduces the chance of alternative sources of contamination in Hilsha fish with *V. cholerae.* This may indicate the traveling of the pathogen with fish to the freshwater aquatic environment from the Bay of Bengal. A limitation of the present study is the lack of the molecular typing data of the isolated *V. cholerae* strains that would strengthen the data to prove the possible transmission route of the reported strains. Further molecular typing experiments, i.e., multilocus sequence typing, next generation sequencing, PFGE, etc. using the *V. cholerae* strains from different sampling points would identify the clonal relationship within the environmental and clinical strains. This study also provides data about producing a high degree of *in vitro* biofilm formation (VPS synthesis) by isolated *V. cholerae* strains from freshly caught Hilsha fish. These findings imply that biofilm plays a part in the persistence of *V. cholerae* during transmission with Hilsha, but further studies are needed to elucidate the proper mechanism and function of *in vivo* biofilm formation and their role in the persistence of *V. cholerae* in the environment. The public health impact of the toxigenic *V. cholerae* strains from Hilsha fish is a matter of concern. People from rural and low-income urban populations with poor access to safe water and sanitation are particularly at risk of *V. cholerae* infection, as proper kitchen hygiene during the cleaning and processing of fish is not regularly maintained. So, proper kitchen hygiene is mandatory during the handling and preparation of Hilsha fish to avoid illness of consumers. The health safety and security of the fishermen during the catching and transportation of the fish to the market should be considered with priority. National policymakers and stakeholders should formulate policies regarding the safe capture, transportation, and storage of fish to ensure the health and safety of the people who earn their livelihood from the fish sector.

## Data availability statement

The original contributions presented in the study are included in the article/Supplementary material, further inquiries can be directed to the corresponding author/s.

## Ethics statement

The animal study was reviewed and approved by the Faculty of Biological Science, University of Dhaka.

## Author contributions

SD designed the research methodology, carried out formal analysis and laboratory work, and prepared the original draft. ZH conceptualized the research method, helped in data curation, and participated in editing the original draft. AB was the principal supervisor of the research and contributed to the revision of the draft, and final approval of the article. PJ participated in a critical reviewing of the original draft. HA helped in the revision and editing of the original draft. All authors have read and agreed to the published version of the manuscript.

## Footnotes

1
http://cope.ku.dk/research/cholera/
