# Supplementary material for: Abundance and biofilm formation capability of Vibrio cholerae in aquatic environment with an emphasis on Hilsha fish (Tenualosa ilisha)
Source: Front Microbiol. 2022 Oct 26;13:933413. doi: 10.3389/fmicb.2022.933413 (PMC9643777; doi:10.3389/fmicb.2022.933413)
Supplement: Supplementary file 1 [file Table_1.DOCX]

**Abundance and Biofilm Formation Capability of *V. cholerae* in Aquatic Environment** **with an Emphasis on Hilsha Fish (*Tenualosa ilisha)***

Subarna Sandhani Dey ^1,2^, Zenat Zebin Hossain ^1,4^, Humaira Akhtar ^1^, Peter K. M. Jensen ^3^ and Anowara Begum ^1*^

^1^Department of Microbiology, University of Dhaka, Dhaka, Bangladesh;

^2^BCSIR Laboratories Rajshahi, Bangladesh Council of Scientific and Industrial Research, Rajshahi-6206, Bangladesh;

^3^Copenhagen Centre for Disaster Research, Institute of Public Health, University of Copenhagen, Denmark;

^4^Department of Public Health, School of Pharmacy and Public Health, Independent University, Bangladesh

*** Correspondence:**Anowara Begum
anowara@du.ac.bd

Supplementary Material

## Supplementary Figures


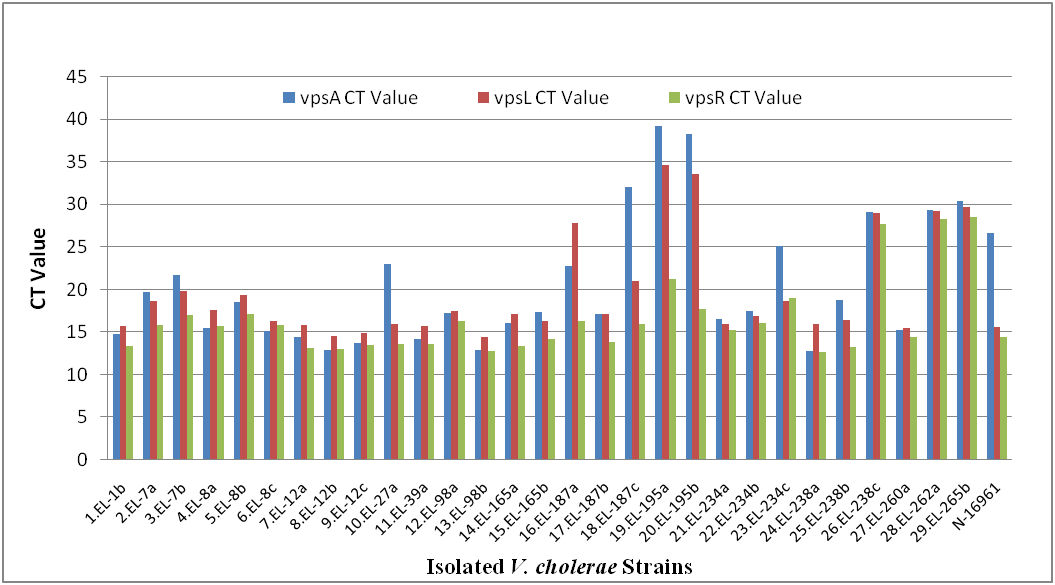


# Supplementary Figure 1. Biofilm formation CT value of vpsA, vpsLand vpsRgene from qPCR.

## Supplementary Tables

**Supplementary Table 1.** Strain ID, serogroup, sampling location, date, presence in different organ

| **Serogroup** | **Strain ID** | **Sampling**  **Location** |  | **Presence in different organ of fish** |  |
| --- | --- | --- | --- | --- | --- |
| **Non-O1,**  **O139** | EL-1(b) | Meghna River,  Gosair Upazilla, Kodalpur |  | Gill |  |
|  | EL-7(a) |  |  | Outer swab |  |
|  | EL-7(b) |  |  | Outer swab |  |
|  | EL-8(a) |  |  | Rectum |  |
|  | EL-8(b) |  |  | Rectum |  |
|  | EL-8(c) |  |  | Rectum |  |
|  | EL-12(a) |  |  | Rectum |  |
|  | EL-12(b) |  |  | Rectum |  |
|  | EL-12(c) |  |  | Rectum |  |
|  | EL-27(a) |  |  | Outer swab |  |
|  | EL-39(a) |  |  | Outer swab |  |
|  | EL-98(a) | Padma River, Dolarchar Union, Kachikata |  | Gut |  |
|  | EL-98(b) |  |  | Gut |  |
|  | EL-165(a) | Padma Rivar, BhedarganjUpazilla |  | Outer swab |  |
|  | EL-165(b) |  |  | Outer swab |  |
|  | EL-187(a) |  |  | Gill |  |
|  | EL-187(b) |  |  | Gill |  |
|  | EL-187(c) |  |  | Gill |  |
|  | EL-195(a) |  |  | Gill |  |
|  | EL-195(b) |  |  | Gill |  |
|  | EL-234(a) | Meghna River,  GosairUpazilla, Kodalpur |  | Gill |  |
|  | EL-234(b) |  |  | Gill |  |
|  | EL-234(c) |  |  | Gill |  |
|  | EL-238(a) |  |  | Gill |  |
|  | EL-238(b) |  |  | Gill |  |
|  | EL-238(c) |  |  | Gill |  |
|  | EL-260(a) |  |  | Outer swab |  |
|  | EL-262(a) |  |  | Gill |  |
|  | EL-265(b) |  |  | Rectum |  |

*no positive sample was found in Tetulia river, Dashmina Upazilla, Bhola and Meghna River, Chandpur.

**Supplementary Table 2.** Sequences of the primers and annealing conditions in the PCRs used for the detection of virulence and regulatory genes

| **Primer name** | **Sequence** | **Annealing Tm (^0^C)** | **Amplicon size (bp)** | **Reference** |
| --- | --- | --- | --- | --- |
| **ompW** | F-CACCAAGAAGGTGACTTTATTGTG | 57 | 304 | (Nandi *et al.*, 2000) |
|  | R-GGTTTGTCGAATTAGCTTCACC |  |  |  |
| **rfb O1** | F-TCTATGTGCTGCGATTGGTG | 57.3 | 638 | (Kumar *et al.*, 2009) |
|  | R-CCCCGAAAACCTAATGTGAG |  |  |  |
| **rfb O139** | F-AGCCTCTTTATTACGGGTGG | 56 | 449 | (Hoshino *et al.*, 1998) |
|  | R-GTCAAACCCGATCGTAAAGG |  |  |  |
| **cep** | F-GCTACATGTTTAGCTCACTG | 48 | 251 | (Chatterjee *et al.*, 2009) |
|  | R-TTTAGCCTTACGAATTAAGCC |  |  |  |
| **tcpA** | F-CGTTGGCGGTCAGTCTTG | 60.3 | 805 | (Kumar *et al.*, 2009) |
|  | R-CGGGCTTTCTTCTTGTTCG |  |  |  |
| **toxR** | F-CCTTCGATCCCCTAAGCAATAC | 52 | 779 | (Singh *et al.*, 2001) |
|  | R-AGGGTTAGCAACGATGCGTAAG |  |  |  |
| **ace** | F-TAAGGATGTGCTTATGATGGACACCC | 55 | 309 | (Kumar *et al.*, 2009) |
|  | R-CGTGATGAATAAAGATACTCATAGG |  |  |  |
| **zot** | F-TCGCTTAACGATGGCGCGTTTT | 52 | 947 | (Kumar *et al.*, 2009) |
|  | R-GTCAAACCCGATCGTAAAGG |  |  |  |
| **ctxB** | F-GGTTGCTTCTCATCATCGAACCAC | 54 | 460 | (Chatterjee *et al.*, 2009) |
|  | R-GATACACATAATAGAATTAAGGAT |  |  |  |
| **tcpI** | 132F-TAG CCT TAG TTC TCA GCA GGC A | 55 | 862 | (Rivera *et al.*, 2001) |
|  | 951R- GGC AAT AGT GTC GAG CTC GTT A |  |  |  |
| **chxA** | F-TGGTGAAGATTCTCCTGCAA | 50 | 421 | (Islam *et al.*, 2013) |
|  | R-CTTGGAGAAATGGATGCGCTG |  |  |  |
| **vasA** | F-GTACGACCGATCCTGACGTT | 58 | 342 | (Hasan *et al.*, 2013) |
|  | R-ATCTGAATGGTCGTGGCTTC |  |  |  |
| **vasK** | F-GCGTCAAATTCAGGAAGAGC | 58 | 399 |  |
|  | R-CTGTCCCAGAACCCAACTGT |  |  |  |
| **vasH** | F-GTGGCACGCTATTTCTGGAT | 57 | 385 |  |
|  | R-TTTCAGCTCACGCACATTTC |  |  |  |
| **msh** | F-AAAAGTCGACAGCGAAAGCGAATAGTGG | 60 | 380 | (Helene Thelin and Taylor, 1996) |
|  | R-AAAAGGATCCATTGCACCAGCAACTGCACC |  |  |  |
| **sxt** | F-ATGGCGTTATCAGTTAGCTGGC | 60 | 1035 | (Bhanumathi *et al.*, 2003) |
|  | R-GCGAAGATCATGCATAGACC |  |  |  |
| **rtxC** | F-CGACGAAGATCATTGACGAC | 52 | 265 | (Chow *et al.*, 2001) |
|  | R-CATCGTCGTTATGTGGTTGC |  |  |  |
| **ompU** | F-CCAAAGCGGTGACAAAGC | 52 | 655 | (Kumar *et al.*, 2009) |
|  | R-TTCCATGCGGTAAGAAGC |  |  |  |
| **hly** | F-GAGCCGGCATTCATCTGAAT | 52 | 480 | (Kumar *et al.*, 2009) |
|  | R-CTCAGCGGGCTAATACGGTTTA |  |  |  |
| **NAG-ST** | F-CCTATTCATTAGCATAATG | 42 | 215 | (Chatterjee *et al.*, 2009) |
|  | R-CCAAAGCAAGCTGGATTGC |  |  |  |
| **vcsN2** | F-CAACACCTTCAAAGCCTTG | 53 | 848 | (Awasthi *et al.*, 2013) |
|  | R-GCGAGCTCCAATTGAAAC |  |  |  |
| **vcsC2** | F-GGTCTCATAGACACTACG | 49 | 589 | (Awasthi *et al.*, 2013) |
|  | R-ACGATGCTATGGGGTATG |  |  |  |
| **vopF** | F-GGAAATTCGCCAAGGTGTA | 49 | 839 | (Awasthi *et al.*, 2013) |
|  | R-CAAAACCGTCCATACAAGG |  |  |  |
| **HA-protease** | F-ACGTTAGTGCCCATGAGGTC | 50 | 350 | (Islam *et al.*, 2013) |
|  | R-ACGGCAAACACTTCAAAACC |  |  |  |

**Supplementary Table 3**. Growth of *V. cholerae* strains at different salt concentrations.

|  | | | | | | |
| --- | --- | --- | --- | --- | --- | --- |
| **Isolated *V. cholerae* strains** | **0% salt** | **3% salt** | **5% salt** | **6% salt** | **8% salt** | **10% salt** |
|  |  |  |  |  |  |  |
| **EL-1b** | 0.013 | 0.289 | 0.274 | 0.034 | 0.031 | 0.021 |
| **EL-7a** | 0.02 | 0.35 | 0.457 | 0.121 | 0.02 | 0.02 |
| **EL-7b** | 0.059 | 0.448 | 0.471 | 0.48 | 0.017 | 0.018 |
| **EL-8a** | 0.018 | 0.372 | 0.668 | 0.723 | 0.024 | 0.012 |
| **EL-8b** | 0.131 | 0.341 | 0.737 | 0.462 | 0.021 | 0.017 |
| **EL-8c** | 0.018 | 0.381 | 0.497 | 0.516 | 0.03 | 0.011 |
| **EL-12a** | 0.028 | 0.276 | 0.2 | 0.152 | 0.027 | 0.022 |
| **EL-12b** | 0.014 | 0.366 | 0.129 | 0.127 | 0.022 | 0.02 |
| **EL-12c** | 0.016 | 0.466 | 0.116 | 0.206 | 0.024 | 0.019 |
| **EL-27a** | 0.003 | 0.546 | 0.174 | 0.133 | 0.029 | 0.021 |
| **EL-39a** | 0.01 | 0.5 | 0.104 | 0.131 | 0.044 | 0.032 |
| **EL-98a** | 0.043 | 0.236 | 0.125 | 0.026 | 0.037 | 0.002 |
| **EL-98b** | 0.051 | 0.091 | 0.115 | 0.064 | 0.023 | 0.003 |
| **EL-165a** | 0.081 | 0.312 | 0.155 | 0.178 | 0.03 | 0.001 |
| **EL-165b** | 0.006 | 0.22 | 0.158 | 0.153 | 0.029 | 0.008 |
| **EL-187a** | 0.128 | 0.241 | 0.251 | 0.302 | 0.025 | 0.025 |
| **EL-187b** | 0.012 | 0.283 | 0.204 | 0.186 | 0.025 | 0.025 |
| **EL-187c** | 0.059 | 0.295 | 0.249 | 0.223 | 0.027 | 0.023 |
| **EL-195a** | 0.084 | 0.427 | 0.418 | 0.325 | 0.037 | 0.026 |
| **EL-195b** | 0.12 | 0.34 | 0.362 | 0.412 | 0.017 | 0.02 |
| **EL-234a** | 0.066 | 0.153 | 0.146 | 0.155 | 0.031 | 0.009 |
| **EL-234b** | 0.058 | 0.315 | 0.376 | 0.32 | 0.03 | 0.013 |
| **EL-234c** | 0.046 | 0.327 | 0.503 | 0.655 | 0.022 | 0.023 |
| **EL-238a** | 0.02 | 0.315 | 0.516 | 0.424 | 0.039 | 0.024 |
| **EL-238b** | 0.015 | 0.18 | 0.165 | 0.482 | 0.04 | 0.006 |
| **EL-238c** | 0.001 | 0.285 | 0.237 | 0.499 | 0.034 | 0.025 |
| **EL-260a** | 0.049 | 0.235 | 0.46 | 0.469 | 0.026 | 0.013 |
| **EL-262a** | 0.03 | 0.466 | 0.278 | 0.505 | 0.133 | 0.012 |
| **EL-265b** | 0.012 | 0.315 | 0.43 | 0.521 | 0.028 | 0.026 |
| **N-16961** | 0.009 | 0.472 | 0.116 | 0.468 | 0.021 | 0.012 |

| **Isolated** **Positive Strains** | **Absorbance 1** | **Absorbance 2** | **Average** | **standard deviation** | **Remarks** |
| --- | --- | --- | --- | --- | --- |
| **Blank** | 0.124 | 0.113 | 0.1185 | 0.007778 | - |
| **EL-1(b)** | 0.602 | 0.626 | 0.614 | 0.016971 | Strong |
| **EL-7(a)** | 0.337 | 0.374 | 0.3555 | 0.026163 | Moderate |
| **EL-7(b)** | 1.33 | 1.367 | 1.3485 | 0.026163 | Strong |
| **EL-8(a)** | 0.302 | 0.386 | 0.344 | 0.059397 | Moderate |
| **EL-8(b)** | 0.381 | 0.442 | 0.4115 | 0.043134 | Moderate |
| **EL-8(c)** | 0.626 | 0.713 | 0.6695 | 0.061518 | Strong |
| **EL-12(a)** | 0.51 | 0.469 | 0.4895 | 0.028991 | Moderate |
| **EL-12(b)** | 0.433 | 0.463 | 0.448 | 0.021213 | Moderate |
| **EL-12(c)** | 0.48 | 0.511 | 0.4955 | 0.02192 | Moderate |
| **EL-27(a)** | 0.235 | 0.284 | 0.2595 | 0.034648 | Weak |
| **EL-39(a)** | 0.481 | 0.542 | 0.5115 | 0.043134 | Moderate |
| **EL-98(a)** | 0.518 | 0.585 | 0.5515 | 0.047376 | Moderate |
| **EL-98(b)** | 0.325 | 0.314 | 0.3195 | 0.007778 | Moderate |
| **EL-165(a)** | 0.299 | 0.224 | 0.2615 | 0.053033 | Weak |
| **EL-165(b)** | 0.226 | 0.208 | 0.217 | 0.012728 | Weak |
| **EL-187(a)** | 0.791 | 0.748 | 0.7695 | 0.030406 | Strong |
| **EL-187(b)** | 0.757 | 0.905 | 0.831 | 0.104652 | Strong |
| **EL-187(c)** | 0.819 | 0.723 | 0.771 | 0.067882 | Strong |
| **EL-195(a)** | 0.439 | 0.638 | 0.5385 | 0.140714 | Moderate |
| **EL-195(b)** | 0.505 | 0.531 | 0.518 | 0.018385 | Moderate |
| **EL-234(a)** | 0.658 | 0.579 | 0.6185 | 0.055861 | Strong |
| **EL-234(b)** | 0.705 | 0.578 | 0.6415 | 0.089803 | Strong |
| **EL-234(c)** | 0.635 | 0.643 | 0.639 | 0.005657 | Strong |
| **EL-238(a)** | 0.477 | 0.681 | 0.579 | 0.14425 | Strong |
| **EL-238(b)** | 0.692 | 0.484 | 0.588 | 0.147078 | Strong |
| **EL-238(c)** | 0.166 | 0.35 | 0.258 | 0.130108 | Moderate |
| **EL-260(a)** | 0.418 | 0.435 | 0.4265 | 0.012021 | Moderate |
| **EL-262(a)** | 0.132 | 0.253 | 0.1925 | 0.08556 | Weak |
| **EL-265(b)** | 0.613 | 0.592 | 0.6025 | 0.014849 | Strong |
| **N-16961** | 0.164 | 0.153 | 0.1585 | 0.007778 | Weak |

**Supplementary Table 4**. Biofilm Formation Ability of Isolated *V. cholerae* strains on Microtiter Plate

References:

Awasthi, S. P. *et al.* (2013) ‘Novel cholix toxin variants, ADP-ribosylating toxins in Vibrio cholerae non-O1/non-O139 strains, and their pathogenicity.’, *Infection and immunity*, 81(2), pp. 531–541. doi: 10.1128/IAI.00982-12.

Bhanumathi, R. *et al.* (2003) ‘Molecular characterization of Vibrio cholerae O139 bengal isolated from water and the aquatic plant Eichhornia crassipes in the River Ganga, Varanasi, India.’, *Applied and environmental microbiology*, 69(4), pp. 2389–2394. doi: 10.1128/AEM.69.4.2389-2394.2003.

Chatterjee, S. *et al.* (2009) ‘Incidence, virulence factors, and clonality among clinical strains of non-O1, non-O139 Vibrio cholerae isolates from hospitalized diarrheal patients in Kolkata, India.’, *Journal of clinical microbiology*, 47(4), pp. 1087–1095. doi: 10.1128/JCM.02026-08.

Chow, K. H. *et al.* (2001) ‘Detection of RTX toxin gene in Vibrio cholerae by PCR’, *Journal of Clinical Microbiology*, 39(7), pp. 2594–2597. doi: 10.1128/JCM.39.7.2594-2597.2001.

Hasan, N. A. *et al.* (2013) ‘Distribution of virulence genes in clinical and environmental Vibrio cholerae strains in Bangladesh.’, *Applied and environmental microbiology*, 79(18), pp. 5782–5785. doi: 10.1128/AEM.01113-13.

Helene Thelin, K. and Taylor, R. K. (1996) ‘Toxin-coregulated pilus, but not mannose-sensitive hemagglutinin, is required for colonization by Vibrio cholerae O1 El Tor biotype and O139 strains’, *Infection and Immunity*, 64(7), pp. 2853–2856. doi: 10.1128/iai.64.7.2853-2856.1996.

Hoshino, K. *et al.* (1998) ‘Development and evaluation of a multiplex PCR assay for rapid detection of toxigenic Vibrio cholerae O1 and O139.’, *FEMS immunology and medical microbiology*, 20(3), pp. 201–207. doi: 10.1111/j.1574-695X.1998.tb01128.x.

Islam, A. *et al.* (2013) ‘Indigenous Vibrio cholerae strains from a non-endemic region are pathogenic’, *Open Biology*, 3(FEB). doi: 10.1098/rsob.120181.

Kumar, P. *et al.* (2009) ‘A large cholera outbreak due to a new cholera toxin variant of the Vibrio cholerae O1 El Tor biotype in Orissa, Eastern India’, *Journal of Medical Microbiology*, 58(2), pp. 234–238. doi: 10.1099/jmm.0.002089-0.

Nandi, B. *et al.* (2000) ‘Rapid method for species-specific identification of Vibrio cholerae using primers targeted to the gene of outer membrane protein OmpW.’, *Journal of clinical microbiology*, 38(11), pp. 4145–4151. doi: 10.1128/JCM.38.11.4145-4151.2000.

Rivera, I. N. G. *et al.* (2001) ‘Genotypes Associated with Virulence in Environmental Isolates of Vibrio cholerae’, *Applied and Environmental Microbiology*, 67(6), pp. 2421–2429. doi: 10.1128/AEM.67.6.2421-2429.2001.

Singh, D. V *et al.* (2001) ‘Molecular analysis of Vibrio cholerae O1, O139, non-O1, and non-O139 strains: clonal relationships between clinical and environmental isolates.’, *Applied and environmental microbiology*, 67(2), pp. 910–921. doi: 10.1128/AEM.67.2.910-921.2001.
